# Sexual Distress in Patients with Hidradenitis Suppurativa: A Cross-Sectional Study

**DOI:** 10.3390/jcm8040532

**Published:** 2019-04-18

**Authors:** Carlos Cuenca-Barrales, Ricardo Ruiz-Villaverde, Alejandro Molina-Leyva

**Affiliations:** 1 Dermatology, Hospital Universitario San Cecilio, Avenida de la Investigación s/n, 18016 Granada, Spain; carloscuenca1991@gmail.com (C.C.-B.); ismenios@hotmail.com (R.R.-V.); 2 Dermatology, Hidradenitis Suppurativa Clinic, Hospital Universitario Virgen de las Nieves, Avenida de las Fuerzas Armadas 2, 18014 Granada, Spain; 3 European Hidradenitis Suppurativa Foundation (EHSF), 06847 Dessau-Roßlau, Germany; 4 Instituto de Investigación Biosanitaria Granada, 18016 Granada, Spain

**Keywords:** sexuality, mental health, mental disorder, sexual dysfunction, hidradenitis suppurativa

## Abstract

Hidradenitis suppurativa (HS) is a chronic auto-inflammatory skin disease with a great impact in quality of life. However, there is little research about the impact of HS on sex life. The aims of this study are to describe the frequency of sexual distress (SD) in patients with HS and to explore potentially associated epidemiological and clinical factors. We conducted a cross-sectional study by means of a crowd-sourced online questionnaire hosted by the Spanish hidradenitis suppurativa patients’ association (ASENDHI). Sexual distress (SD) was evaluated with a Numeric Rating Scale (NRS) for HS impact on sex life. A total of 393 participants answered the questionnaire. The mean NRS for HS impact on sex life was 7.24 (2.77) in women and 6.39 (3.44) in men (*p* < 0.05). Variables significantly associated (*p* < 0.05) with SD in the multiple linear regression model were sex, with a higher risk in females, the presence of active lesions in the groin and genitals and NRS for pain and unpleasant odor; being in a stable relationship was an important protector factor. Regarding these results, it seems that SD in HS patients is due, at least in part, to disease symptoms and active lesions in specific locations, emphasizing the importance of disease control with a proper treatment according to management guidelines. Women and single patients are more likely to suffer from sexual distress.

## 1. Introduction

Hidradenitis suppurativa (HS) is a chronic auto-inflammatory skin disease characterized by recurrent nodules, abscesses and fistulae and which involves hair follicles, predominantly in intertriginous areas [1]. These lesions cause pain, unpleasant odor, itching and suppuration. When the disease progresses to advanced stages, there may be a permanent negative effect on body image due to scarring.

According to recent studies, the reduction in HS patients’ quality of life is one of the most significant among dermatological patients [2,3] and similar to other non-dermatological illnesses such as chronic obstructive pulmonary disease, diabetes mellitus, cardiovascular disease and cancer [4]. Some research indicates that pain or pruritus may negatively affect quality of life [5,6].

Sexuality is a basic need and one which cannot be separated from other aspects of human life, being extremely important for maintaining good mental health [7]. Several studies show a direct relationship between sexual function and quality of life [8,9]. Sexual functionality can be impaired by chronic diseases because of factors related to the disease itself, its treatments, or alterations in body image [10]. Due to the chronic relapsing course of HS and the disease’s characteristics, HS may affect patients’ sexuality. Numerous publications have associated HS with depression, anxiety, low self-esteem, loneliness, stigmatization, suicide risk, or impact on working life [2,3,11,12,13,14,15,16]. However, there is little research about the impact of HS on sex life.

The aims of this study are to describe the frequency of sexual distress (SD) in patients with HS and to explore potentially associated epidemiological and clinical factors.

## 2. Experimental Section

### 2.1. Patients and Design

We conducted a cross-sectional study by means of a crowd-sourced online questionnaire. Participants were recruited from 1 March to 1 April 2018. The Spanish hidradenitis suppurativa patients’ association (ASENDHI) hosted the questionnaire and invited people with HS to participate in the study [17].

The selection criterion was self-referred diagnosis of HS. Participants were aware of the questionnaire’s anonymity and the use of their data for research purposes. The study was approved on May 2017 by the ethics committee of Hospital Universitario San Cecilio and is in accordance with the World Health Organization Declaration of Helsinki.

### 2.2. Questionnaire

The questionnaire was developed with Google^®^ Forms suite. Socio-demographic data, biometric parameters, use of medication for other comorbidities and several characteristics of the disease, such as age of onset, time under medical attention and affected areas were collected. Disease severity was assessed by patients’ self-reported Hurley stage, since patients with HS are capable of self-assessing their Hurley stage with a good correlation with physician assessment [18].

Disease activity was assessed by Patients’ Global Assessment (PtGA), including five categories (inactive, very low, low, mild and severe) [19], and intensity of symptoms by Numeric Rating Scales (NRS) [20]. These scales show the subjective impact of the disease on patients, with equal or greater importance than objective scales.

SD was evaluated with a NRS for HS impact on sex life, in which participants were asked to measure from 0 to 10 how much the disease affects their sex life. This scale reflects the subjective suffering and distress caused by the disease to patients’ sex lives. Its concordance with the Female Sexual Function Index-6 (FSFI-6) and the International Index of Erectile Function-5 (IIEF-5), two validated questionnaires that explore female sexual dysfunction and erectile dysfunction respectively, was also assessed.

### 2.3. Statistical Analysis

Statistical analyses were performed using JMP version 9.0.1 (SAS institute, Inc., Cary, NC, USA). When there were missing data in any of the variables of interest, patients were excluded from the study. When missing data were found in other variables, they were imputed. To explore the characteristics of the sample, descriptive statistics were used. Continuous variables were expressed as means and standard deviations. Qualitative variables were expressed as absolute and relative frequencies.

The main outcome of interest was SD, measured by the NRS for HS impact on sex life. To explore possibly associated factors, simple linear regression was used for continuous variables, Student’s t-test for dichotomous variables, and one-way analysis of variance for nominal variables with two or more categories (Levene’s test was used to assess the equality of variances, standardized residual plots to check independence and Normality was assumed because of the sample size). Significantly associated variables (*p* < 0.05) or those showing trends towards statistical significance (*p* < 0.20) were included in a multiple linear regression model to assess the factors associated with SD. Statistical significance was considered if *p* values were less than 0.05.

The correlation of NRS for HS impact on sex life with FSFI-6 and IIEF-5 was checked with simple linear regression. Student’s *t*-test was used to assess differences between NRS for HS impact in sex life means in participants with and without sexual or erectile dysfunction according to the FSFI-6 or IIEF-5 scores, respectively. The cut-off point for sexual dysfunction using the NRS for HS impact on sex life was assessed by ROC curve analysis.

## 3. Results

### 3.1. Baseline

Three hundred and ninety three participants answered the questionnaire. Seven of them filled out the questionnaire incompletely, so the final sample consisted of 386 participants (319 (82.6%) from Spain, 57 (14.8%) from abroad, and 10 (2.6%) did not provide their country of residence). The ratio of women to men was 3.8:1 (306 (79.27%) women and 80 (20.73%) men). Their socio-demographic characteristics and comorbidities are shown in Table 1; current smoking was higher among men, body mass index was 1.5 greater in women, and the prevalence of diabetes mellitus type II and antidepressant consumption was higher among women, but these differences did not reach statistical significance. HS baseline characteristics are shown in Table 2. Age of onset was earlier in women (19.09 ± 7.1 vs. 23.57 ± 9.45, *p* < 0.0001), with a medium diagnosis delay of 11.23 ± 9.55 in women and 8.86 ± 9.13 in men. The groin was the location most affected in women, either by active lesions (65.7%) or scars (57.2%). In men, groin was the location more frequently affected by active lesions (53.8%), and axilla by scars (47.5%). Genitals were affected by active lesions in 111 (36.3%) of women and in 31 (38.8%) of men, and by scars in 82 (26.8%) of women and in 28 (35%) of men. The presence of active lesions in the perianal region (35 (43.8%) vs. 50 (16.3%), *p* < 0.0001) and on the buttocks (35 (43.8%) vs. 95 (31%), *p* <0.05) were higher among men, while the breast region was more frequently affected in women (90 (29.4%) vs. 2 (2.5%), *p* < 0.0001).

### 3.2. Sexual Distress and Related Factors in Patients with Hidradenitis Suppurativa

The mean NRS for HS impact on sex life was 7.24 (2.77) in women and 6.39 (3.44) in men (*p* < 0.05). Results from univariate analysis of factors possibly related to NRS for HS impact on sex life are shown in Table 3.

Variables that were significantly associated or showed trends towards statistical significance (*p* < 0.20) were included in the multiple linear regression model, whose results are shown in Table 4. Variables significantly associated with SD were sex, with a higher risk in females, the presence of active lesions in the groin and genitals and NRS for pain and unpleasant odor; being in a stable relationship was an important protector factor for SD. Current smoking, PtGA, time under medical attention and treatment with adalimumab showed trends toward statistical significance.

### 3.3. Correlation between NRS for HS Impact on Sex Life and FSFI-6/IIEF-5 Scores

Scores from NRS for HS impact on sex life and FSFI-6 showed a negative correlation (β = −0.15 ± 0.02, *r*^2^ = 0.16, *p* < 0.0001), indicating a good concordance between both questionnaires. Scores from NRS for HS impact on sex life and IIEF-5 also showed a negative correlation (β = −0.21 ± 0.05, *r*^2^ = 0.15, *p* < 0.001). The mean score on the NRS for HS impact on sex life was 8.27 ± 0.21 in women with sexual dysfunction, and 6.16 ± 0.21 in women without sexual dysfunction (*p* < 0.0001). In men, the mean score on the NRS for HS impact on sex life was 7.31 ± 0.47 in those with erectile dysfunction, and 5 ± 0.58 in those without erectile dysfunction (*p* < 0.01).

**Figure 1:**
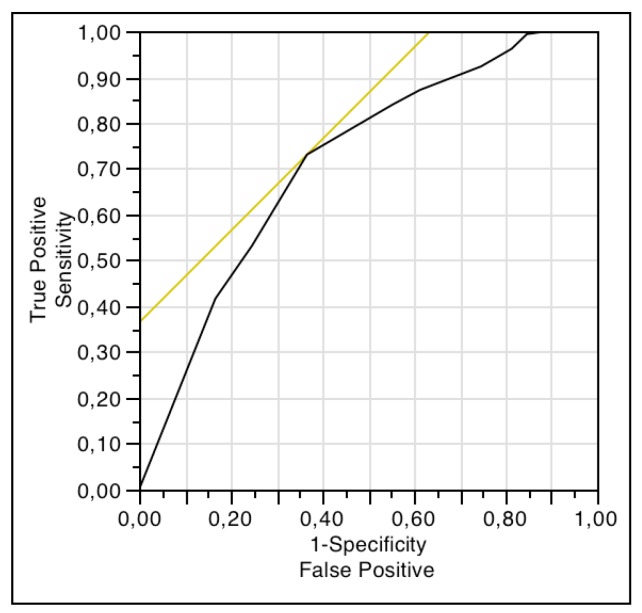
ROC curve analysis for comparison between scores of NRS of HS impact on sex life and FSFI-6.

**Figure 2:**
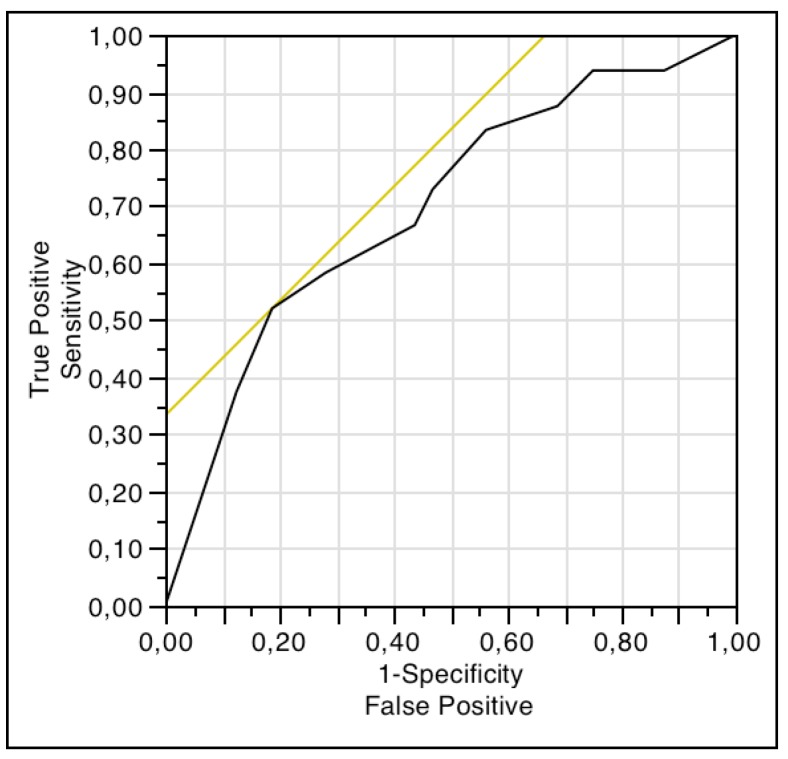
ROC curve analysis for comparison between scores of NRS of HS impact on sex life and IIEF-5.

In women, a score of 8 or more on the NRS for HS impact on sex life was indicative of sexual dysfunction according to FSFI-6 scores, with a sensitivity of 73% and a specificity of 64% (Figure 1). In men, a score of 9 or more on the NRS for HS impact on sex life was indicative of erectile dysfunction according to IIEF-5 scores, with a sensitivity of 52% and a specificity of 81% (Figure 2).

## 4. Discussion

To our best knowledge, this is the largest cross-sectional study about the impact of HS on sexuality. Socio-demographic and disease characteristics did not differ from those previously reported in the literature, and were representative of the general HS population [21,22,23,24,25,26,27,28].

The mean NRS score for HS impact on sex life was significantly higher in women, which tallies with previous research that indicates higher sexual distress in women than in men with HS [29] or psoriasis [30]. These differences have been associated with cultural aspects and differences in emotional and neuroendocrine responses to disfigurement, and with the earlier onset of HS in women (4.5 years earlier in our sample) [29]. A higher prevalence of lesions at the lower abdomen has also been posed as a reason for this higher distress in women [29], but in our sample we only observed more involvement below the abdomen in the groin.

Although in psoriasis the involvement of the anogenital area has been related to sexual dysfunction [31,32,33], in HS anogenital involvement has been related to a reduction in quality of life [2,34], but there are no locations related to sexual dysfunction or to sexual distress [29,34]. In our investigation, we found an association between active lesions in the groin and genitals and SD, so a properly medical/surgical intervention at this level could turn into a better sexual life. In previous research about sexual health in patients with HS, samples were taken from hospital departments [29,34] and from a patient’s association [34], and there were no important differences in patients’ baseline characteristics, with the exception of a more prevalent Hurley III stage in our sample. Therefore, these findings were probably made possible due to the larger size of our sample.

Moreover, subjective symptoms caused sexual distress. The intensity of pain and unpleasant odor were related with higher scores on NRS for HS impact on sex life. This association may be due to factors directly related to the nature of the sexual act and/or to psychological factors that could be related to disease activity [2], highlighting the importance of symptom management to improve sexual health in patients with HS. Other factors such as antidepressant or benzodiazepine use were not statistically associated with SD, suggesting that SD is directly related to organic symptoms.

The absence of a stable relationship was not associated with sexual dysfunction in previous research [29,34]. Nevertheless, we observed that the presence of a stable partner was importantly related to lower SD. Since having a partner is associated with less self-consciousness and less orgasm difficulty in both men and women [35], probably feelings of shame, distrust, shyness and rejection influence SD, which could be lessened by the trust built in a relationship.

There were other factors that showed trends toward statistical significance in the multiple linear regression model: (1) PtGA, pointing to the importance of disease activity in sexual distress and the need to control the inflammatory load; (2) current smoking, because it is related to greater disease activity, since it favors follicular occlusion, a proinflammatory state with activation of neutrophils and Th17 lymphocytes, induces biofilm formation and suppresses notch signaling, among other effects [36]; moreover, smoking cessation is associated with clinical improvement [37]; (3) time under medical care, since it reflects time of disease evolution, with cumulative life course impairment [38]; and (4) Treatment with adalimumab, probably because in our sample it is a better predictor of severity than Hurley, since the patients treated with adalimumab are the most severe.

Finally, we found a good correlation between the scores on NRS for HS impact on sex life and those of FSFI-6 and IIEF-5, which indicates an association between the subjective and objective involvement of the sexual sphere in participants. However, despite this concordance, the ROC curve analyses revealed that the NRS for HS impact on sex life was not a good tool to assess sexual dysfunction in women or erectile dysfunction in men, because the cut-off points reach neither an acceptable sensitivity nor specificity. It is important to the clinician to distinguish between sexual distress and sexual dysfunction, because the first reveals the suffering of the subject, whereas the second may mean a worse sexual experience for both members of the stable/sporadic relationship. Therefore, clinicians should assess both aspects when patients’ sexuality is addressed.

There are some methodological weaknesses in our study: (1) A possible selection bias, since it only represents patients in contact with support groups and Internet access. The elderly, who may use the Internet less frequently, or those with low sociocultural status or fear of new technologies, could be under-represented [39]. Moreover, people already concerned about sexual problems may have been more likely to answer the questionnaire. Nonetheless, the baseline characteristics of our sample did not differ from those previously reported in the literature, either in hospital-based or population-based studies. Given the scarcity of information about HS and sexuality we consider that this study is a good introduction to the problem, and could lay the foundation for future research. (2) A possible classification bias, because it was an online questionnaire and HS diagnosis could not be confirmed; HS characteristics were also self-referred. Nevertheless, an informed population can properly identify HS, because of its apparent and distinctive clinical manifestations. Since a patients’ association hosted the questionnaire, it is expected that the participants did suffer from the disease.

## 5. Conclusions

This is the largest cross-sectional study about HS and sexuality. We have observed important sexual distress in patients with HS. Factors related to SD were female sex, the presence of active lesions in the groin and genital areas, and the intensity of pain and unpleasant odor. Being in a stable relationship has been an important protector factor against SD. Regarding these results, it seems that SD in HS patients is due, at least in part, to disease symptoms and active lesions in specific locations, emphasizing the importance of proper control of the disease based on management guidelines to improve their sexual health. Women and single patients are more likely to suffer from sexual distress, so special medical care should be given to them.

## Figures and Tables

**Table 1:** Socio-demographic characteristics and comorbidities.

	Men (*n* = 80)	Women (*n* = 306)	All (*n* = 386)
Age	39.21 ± 11.15	37.44 ± 8.69	37.81 ± 9.26
BMI	28.12 ± 5.03	29.67 ± 7.05	29.35 ± 6.71
Current smoker			
No	28 (35%)	135 (44.1%)	163 (42.2%)
Yes	52 (65%)	171 (55.9%)	223 (57.8%)
Comorbidities			
HBP	4 (5%)	21 (6.9%)	25 (6.5%)
DM2	2 (2.5%)	20 (6.5%)	22 (5.7%)
Dyslipidemia	3 (3.8%)	9 (2.9%)	12 (3.1%)
Antidepressant use	4 (5%)	31 (10.1%)	35 (9.1%)
Benzodiazepine use	4 (5%)	18 (5.9%)	22 (5.7%)
Stable relationship	54 (67.5%)	236 (77.1%)	290 (75.1%)

Continuous variables are expressed as means ± standard deviation and qualitative variables as absolute (relative) frequencies. BMI: body mass index. HBP: high blood pressure. DM2: diabetes mellitus type 2.

**Table 2:** Hidradenitis suppurativa (HS) patients’ baseline characteristics.

	Men (*n* = 80)	Women (*n* = 306)	All (*n* = 386)
Time of evolution	15.64 ± 10.53	18.33 ± 9.3	17.77 ± 9.62
Time under medical attention	6.79 ± 7.21	7.1 ± 7.29	7.03 ± 7.27
Number of active regions	2.73 ± 1.79	2.5 ± 1.57	2.55 ± 1.62
Number of regions with scars	2.34 ± 2.29	2.31 ± 2.06	2.31 ± 2.1
Hurley state			
I	13 (16.3%)	55 (18%)	68 (17.6%)
II	25 (31.3%)	149 (48.7%)	174 (45.1%)
III	42 (52.5%)	102 (33.3%)	144 (37.3%)
PtGA	3.73 ± 1.04	3.65 ± 1.11	3.66 ± 1.09
NRS pain	6.64 ± 2.81	6.52 ± 2.98	6.54 ± 2.95
NRS pruritus	6.24 ± 2.67	6.48 ± 3.03	6.43 ± 2.96
NRS unpleasant odor	6.11 ± 3.05	5.47 ± 3.45	5.6 ± 3.38
NRS suppuration	6.84 ± 3.04	6.39 ± 3.21	6.48 ± 3.18

Continuous variables are expressed as means ± standard deviation and qualitative variables as absolute (relative) frequencies. PtGA: Patient’s Global Assessment; values range from 1 (inactive disease) to 5 (severe disease). NRS: Numeric Rating Scale; values range from 0 (no symptoms) to 10 (maximum intensity of symptoms).

**Table 3:** Univariate analysis of factors associated with sexual distress in patients with HS.

KERRYPNX	Univariate Analysis	*p*-Value
Sex		0.021 *
Female	$\bar{x}$ = 7.24 (0.17)	
Male	$\bar{x}$ = 6.39 (0.33)	
Age	β = −0.01 (0.02)	0.738
Current smoker		0.023 *
Yes	$\bar{x}$ = 7.35 (0.2)	
No	$\bar{x}$ = 6.66 (0.23)	
Antidepressant use		0.51
Yes	$\bar{x}$ = 7.37 (0.5)	
No	$\bar{x}$ = 7.03 (0.16)	
Benzodiazepine use		0.692
Yes	$\bar{x}$ = 6.82 (0.63)	
No	$\bar{x}$ = 7.07 (0.15)	
Age of onset	β = −0.01 (0.02)	0.667
Time under medical attention	β = 0.04 (0.02)	0.042 *
Active lesions in axilla		0.532
Yes	$\bar{x}$ = 7.16 (0.22)	
No	$\bar{x}$ = 6.97 (0.21)	
Scars in axilla		0.607
Yes	$\bar{x}$ = 7.15 (0.22)	
No	$\bar{x}$ = 6.99 (0.2)	
Active lesions in groin		<0.0001 *
Yes	$\bar{x}$ = 7.63 (0.18)	
No	$\bar{x}$ = 6.09 (0.24)	
Scars in groin		0.169
Yes	$\bar{x}$ = 7.25 (0.2)	
No	$\bar{x}$ = 6.84 (0.22)	
Active lesions on genitals		<0.0001 *
Yes	$\bar{x}$ = 7.99 (0.24)	
No	$\bar{x}$ = 6.16 (0.18)	
Scars on genitals		0.022 *
Yes	$\bar{x}$ = 7.6 (0.28)	
No	$\bar{x}$ = 6.84 (0.18)	
Active lesions on buttocks		0.065
Yes	$\bar{x}$ = 7.45 (0.26)	
No	$\bar{x}$ = 6.86 (0.18)	
Scars on buttocks		0.566
Yes	$\bar{x}$ = 6.94 (0.26)	
No	$\bar{x}$ = 7.12 (0.18)	
Active lesions on breast		0.026 *
Yes	$\bar{x}$ = 7.65 (0.3)	
No	$\bar{x}$ = 6.87 (0.17)	
Scars on breast		0.327
Yes	$\bar{x}$ = 7.33 (0.31)	
No	$\bar{x}$ = 6.98 (0.17)	
Active lesions on abdomen		0.219
Yes	$\bar{x}$ = 7.6 (0.46)	
No	$\bar{x}$ = 7 (0.16)	
Scars on abdomen		0.77
Yes	$\bar{x}$ = 7.18 (0.44)	
No	$\bar{x}$ = 7.04 (0.16)	
Active lesions in perianal region		0.144
Yes	$\bar{x}$ = 7.47 (0.32)	
No	$\bar{x}$ = 6.94 (0.17)	
Scars in perianal region		0.168
Yes	$\bar{x}$ = 7.46 (0.33)	
No	$\bar{x}$ = 6.95 (0.17)	
Active lesions on neck		0.805
Yes	$\bar{x}$ = 7.2 (0.59)	
No	$\bar{x}$ = 7.05 (0.16)	
Scars on neck		0.791
Yes	$\bar{x}$ = 7.22 (0.61)	
No	$\bar{x}$ = 7.05 (0.15)	
Number of regions with active lesions	β = 0.48 (0.09)	<0.0001 *
Number of regions with scars	β = 0.1 (0.07)	0.182
Hurley stage		0.01 *
I	$\bar{x}$ = 6.21 (0.35)	
II	$\bar{x}$ = 7.02 (0.22)	
III	$\bar{x}$ = 7.51 (0.24)	
Treatment with oral antibiotics		0.074
Yes	$\bar{x}$ = 7.48 (0.28)	
No	$\bar{x}$ = 6.89 (0.18)	
Treatment with oral contraceptives		0.833
Yes	$\bar{x}$ = 7.13 (0.38)	
No	$\bar{x}$ = 7.05 (0.16)	
Treatment with adalimumab		0.03 *
Yes	$\bar{x}$ = 7.82 (0.38)	
No	$\bar{x}$ = 6.92 (0.16)	
PtGA	β = 0.87 (0.13)	<0.0001 *
NRS for pain	β = 0.32 (0.05)	<0.0001 *
NRS for pruritus	β = 0.27 (0.05)	<0.0001 *
NRS for unpleasant odor	β = 0.25 (0.04)	<0.0001 *
NRS for suppuration	β = 0.25 (0.05)	<0.0001 *
Stable relationship		0.032 *
Yes	$\bar{x}$ = 6.88 (0.17)	
No	$\bar{x}$ = 7.62 (0.3)	

*p*-values of variables significantly associated are marked with * PtGA: Patient’s Global Assessment; values range from 1 (inactive disease) to 5 (severe disease). NRS: Numeric Rating Scale; values range from 0 (no symptoms) to 10 (maximum intensity of symptoms).

**Table 4:** Multivariate analysis of factors associated with sexual distress in patients with HS.

	Multivariate Analysis	*p*-Value
Sex (female)	β = 0.57 (0.19)	0.003 *
Current smoker	β = 0.27 (0.14)	0.059
Time under medical attention	β = 0.03 (0.02)	0.088
Active lesions in groin	β = 0.44 (0.18)	0.015 *
Scars in groin	β = 0.15 (0.19)	0.449
Active lesions on genitals	β = 0.4 (0.19)	0.033 *
Scars on genitals	β = 0.05 (0.21)	0.812
Active lesions on buttocks	β = 0.19 (0.18)	0.296
Active lesions on breast	β = 0.09 (0.21)	0.666
Active lesions in perianal region	β = 0.15 (0.21)	0.463
Scars in perianal region	β = 0.23 (0.21)	0.28
Number of regions with active lesions	β = 0.15 (0.19)	0.44
Number of regions with scars	β = 0.14 (0.12)	0.24
Hurley stage		
III vs. I	β = 0.07 (0.26)	0.804
III vs. II	β = 0.03 (0.19)	0.866
Treatment with oral antibiotics	β = 0.02 (0.16)	0.9
Treatment with adalimumab	β = 0.38 (0.2)	0.054
PtGA	β = 0.3 (0.19)	0.115
NRS for pain	β = 0.15 (0.08)	0.049 *
NRS for pruritus	β = 0.03 (0.06)	0.615
NRS for unpleasant odor	β = 0.13 (0.06)	0.035 *
NRS for suppuration	β = 0.05 (0.07)	0.489
Stable relationship	β = −0.56 (0.16)	<0.001 *

*p* values of variables significantly associated are marked with * PtGA: Patient’s Global Assessment; values range from 1 (inactive disease) to 5 (severe disease). NRS: Numeric Rating Scale; values range from 0 (no symptoms) to 10 (maximum intensity of symptoms).
